# Practice Transformation Driven through Academic Partnerships

**DOI:** 10.3390/pharmacy8030120

**Published:** 2020-07-14

**Authors:** Renee Robinson, Cara Liday, Anushka Burde, Tracy Pettinger, Amy Paul, Elaine Nguyen, John Holmes, Megan Penner, Angela Jaglowicz, Nathan Spann, Julia Boyle, Michael Biddle, Brooke Buffat, Kevin Cleveland, Brecon Powell, Christopher Owens

**Affiliations:** 1 Department of Pharmacy Practice and Administrative Sciences, College of Pharmacy, Idaho State University, Anchorage Campus, Anchorage, AK 99508, USA; paulamy@isu.edu (A.P.); pennmeg2@isu.edu (M.P.); jaglange@isu.edu (A.J.); 2 Department of Pharmacy Practice and Administrative Sciences, College of Pharmacy, Idaho State University, Pocatello Campus, Pocatello, ID 83209, USA; lidacara@isu.edu (C.L.); burdanu1@isu.edu (A.B.); petttra1@isu.edu (T.P.); holmjohn@isu.edu (J.H.); bbuffat@isu.edu (B.B.); powebrec@isu.edu (B.P.); owenchri@isu.edu (C.O.); 3 Department of Pharmacy Practice and Administrative Sciences, College of Pharmacy, Idaho State University, Meridian Campus, Meridian, ID 83642, USA; nguyelai@isu.edu (E.N.); spannath@isu.edu (N.S.); boyljuli@isu.edu (J.B.); biddmich@isu.edu (M.B.); clevkevi@isu.edu (K.C.)

**Keywords:** primary health care, pharmacy, evidence-based pharmacy practice, health outcomes, academic, dissemination, practice transformation, implementation science, quality improvement

## Abstract

Evidence-based interventions have been shown to improve the quality of patient care, reduce costs, and improve overall health outcomes; however, adopting new published research and knowledge into practice has historically been slow, and requires an active, systematic approach to engage clinicians and healthcare administrators in the required change. Pharmacists have been identified as important agents of change and can enhance care delivery in primary care settings through evidence-based interventions. Utilizing the Consolidated Framework for Implementation Research (CFIR) we identify, assess, and share barriers and facilitators to program development, as well as growth and expansion efforts across five discrete, university-subsidized, embedded-pharmacy practices in primary care. We identified two overarching modifiable factors that influence current and future practice delivery and highlight the role of academia as an incubator for practice change and implementation: Data collection and information sharing. Conceptual frameworks such as CFIR help establish a common vernacular that can be used to facilitate systematic practice site implementation and dissemination of information required to support practice transformation.

## 1. Introduction

Evidence-based interventions have been shown to improve the quality of care, reduce costs, and improve health and humanistic outcomes [1,2]. However, adopting new published research and knowledge into practice has historically been slow [3,4]. To increase the type and amount of practice-based evidence to drive innovation and subsequently evidence-based practice in all patient care settings, a better support for systematic engagement is needed. Academic pharmacists in clinical teaching and patient care roles are ideally situated to identify and address barriers to implementation, information dissemination, and to establish mechanisms to support lasting practice change.

Pharmacists holding faculty positions (i.e., academic pharmacists) who are embedded in clinical settings have the responsibility of providing direct patient care in collaboration with interprofessional teams as well as facilitating clinical learning experiences for pharmacy students and residents [5]. Such positions may be in healthcare systems, hospitals, primary care clinics, community pharmacies, or other practice settings. In addition to patient care activities and teaching, academic pharmacists often have scholarship responsibilities and are expected to disseminate the results of innovative practice activities. The American Medical Association (AMA) has called for embedding pharmacists into practice as a means of enhancing patient care, raising physician satisfaction, and supporting practice sustainability [6]. In these settings, pharmacists are already driving change in implementation science.

Dissemination and implementation (DI) science is a relatively new discipline that provides a framework for stakeholders (i.e., researchers, clinicians, and healthcare administrators) to identify, interpret, evaluate, and disseminate evidence-based research findings into practice (implementation) [3]. DI intends to bridge the gap between research and practice, translating evidence-based practice and research into real-world settings using conceptual frameworks and translating lessons learned into strategies that fit the daily workflow of a variety of clinical settings. Conceptual frameworks such as those used in DI increase generalizability and interpretability of results, and expedite the application of findings into practice by highlighting factors known to influence the outcomes of interest in this case, implementation of interventions to improve health care delivery by better utilization of pharmacists in the primary care setting.

DI has been used by pharmacists to study practice advancement and implementation in a variety of settings (e.g., community pharmacies in Spain, hospital pharmacies in the US and United Kingdom) [7,8,9]. However, to our knowledge it has not been used to explore the barriers and facilitators of change pharmacists face when embedded in primary care settings and specifically the role academic pharmacists can play in this incubator of change. There are many DI tools, frameworks, and logic models (project roadmaps) available to assist stakeholders and guide systematic evaluation. The Consolidated Framework for Implementation Research (CFIR) is a construct widely used in health services and pharmacy research for over 20 years [10]. CFIR is intended to be flexible, enabling researchers to tailor the framework to the specific intervention design, factors, and context studied.

In this article, we utilized CFIR and examples from five university-subsidized academic pharmacists to identify and assess barriers and facilitators to the initiation and expansion of embedded-pharmacy practice in primary care. The analysis focused on sharing the embedded pharmacist’s experiences with practice implementation, steps taken, and examples of how these changes impacted healthcare delivery and practice at their site (e.g., enhanced medication adherence, reduced adverse events, and improved patient satisfaction). We hope through shared experience in a structured format (CFIR) to guide pharmacists in the US and other countries to support program development and expansion of pharmacist non-dispensing services in primary care.

## 2. Materials and Methods

To present our approach, we utilize a case series format, informed by a formative cross-site, qualitative investigation of five sites in which the pharmacist was embedded in a primary care setting. All sites were supported by one university system, Idaho State University. Partner healthcare facilities varied in size, organization type (non-profit, for-profit), population served, and geographic location. The practice transformation initiative spanned two rural and frontier states that historically have limited healthcare resources (Idaho and Alaska).

**Figure 1:**
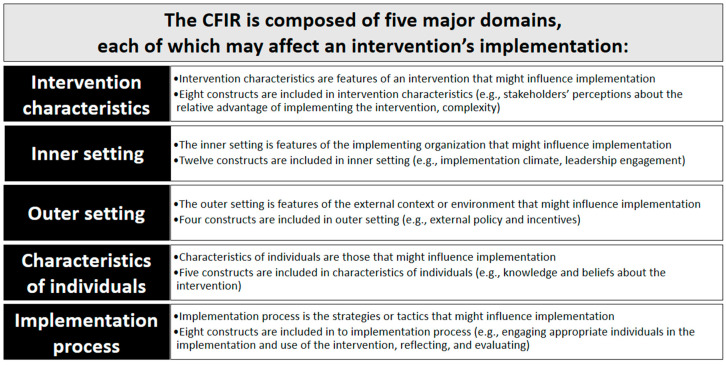
Consolidated Framework for Implementation Research (CFIR) domains.

To understand how practice sites experienced and/or were experiencing implementation changes we developed a semi-structured interview guide, scheduled and interviewed pharmacists within the embedded practice site(s). The interview guide focused on the factors surrounding practice transformation and asked embedded pharmacists at the five sites to: (1) Describe the changes made by primary care practice sites to embed the pharmacist faculty within the practice, (2) identify barriers and facilitators to implementation, and (3) share personal experiences of how the embedded pharmacist has engaged in the primary care practice. Questions focused on: (1) How the embedded pharmacist was operationalized in the primary care setting including exploration and installation steps, (2) how the existing practice and workflow functions did or did not support the embedded pharmacist, (3) what was helpful or not helpful during the implementation process, and (4) the pharmacists’ perception of how other stakeholders (patients, providers, and the healthcare systems) viewed their addition to the healthcare team. A template analysis approach was used to code interviews with individual embedded pharmacists. As part of the process, codes were refined, coding definitions established, coding rules developed, and interviews coded. CFIR domains and constructs were used to contextualize findings that represented the factors influencing embedded pharmacists’ implementation in primary care (Figure 1) [11]. Coded reports were then used to identify whether the finding (and matched construct) exerted a negative, positive, or neutral influence on implementation.

A narrative report of barriers and facilitators identified by site was developed by the desired patient outcome (e.g., improved clinical outcomes, increased medication adherence, and reduced adverse drug events) and was linked to the Centers for Medicare and Medicaid Services (CMS) established core primary care (CPC) functions. CPC functions include insights on practice readiness, care delivery and redesign, actionable performance-based incentives, necessary health information technology (HIT), and data sharing. The five core CPC functions are: (1) Risk-stratified care management, (2) access and continuity, (3) planned care for chronic conditions and preventive care, (4) patient and caregiver engagement, and (5) coordination of care across the medical neighborhood. Similarities, differences, and trends in how the practice sites experienced change were reviewed and summarized. Drawing on the analytic matrices for each program component and CFIR domain, summary tables were developed to visualize barriers and facilitators and support identification of key areas where additional support would be necessary for long-term sustainability.

## 3. Results

What happened during the embedded pharmacists’ implementation? How did various constructs influence operationalization of primary care workflows? What was the impact of the embedded pharmacist on patient clinical outcomes, medication adherence, adverse drug events, and patient satisfaction? The important contextual factors and examples as they relate to practice site implementation, operationalization, and outcomes experienced at the five practice sites are shared below. In Table 1, select factors contributing to perceived readiness (established from interview data) were organized by CFIR domains and CPC components. In Table 2, barriers and facilitators to implementation are presented.

Clinical outcomes: In 1998, a university-sponsored embedded pharmacist position was established at a for-profit, family practice clinic. This non-dispensing clinical pharmacy position, one of the first of its kind in Idaho, was created as a rotation site for students to complete required advanced pharmacy practice experiences (APPE) in primary care and was fully subsidized by the university.

Initially foreign to patients, providers, and healthcare administrators, it took approximately six years to garner the necessary trust of healthcare providers and administrators for the pharmacist to begin taking on specific tasks/roles to support the clinic providers (e.g., conducting chart reviews, managing anticoagulation therapy). Relationships between the embedded pharmacist and clinic developed over a six-year period starting with collaborating with providers to suggest evidence-based pharmacotherapy recommendations to better meet clinic patient needs. Later, case examples and individual-level health outcome data resulting directly from the embedded pharmacist contributions were shared with providers and healthcare administrators. Improvements in traditional documented health outcomes such as hemoglobin A1c and satisfaction metrics were shared with providers and healthcare teams to demonstrate impact. It is noteworthy that, it was not until the embedded pharmacist became a Certified Diabetes Educator (CDE) that healthcare providers and administrators within the system more fully understood her role as a billable healthcare service provider supporting the combined clinic visit structure. Initially, the primary care practice only supported using billing codes such as 99211 typically used for the evaluation and management of an established patient with minimal presenting problem(s) addressed in approximately 5 min, underbilling for pharmacist time, and health services provided. Over the next 10 years, formal reimbursement and collaborative practice agreements were established with the healthcare facility to enable the embedded pharmacist to independently bill for the non-dispensing services provided and for the pharmacist outcome metrics to be reported with system health metrics to insurers.

In 2007, the university approached a physician-owned clinic to establish another university-subsidized, embedded-pharmacist position within primary care. The physician-owned clinic, located in southeastern Idaho, provided care to rural communities and offered a unique opportunity for the academic pharmacists, along with pharmacy residents and students to identify and address rural health concerns of rural patients. Some clinical providers in this clinic were previously exposed to pharmacy residents and clinical pharmacy services, but were not familiar with all of the available supports an embedded pharmacist could provide within a primary care clinic. It was based on this experience that an embedded position was created.

Over the following 13 years, the scope of pharmacy practice expanded to include management of all health conditions covered within the clinic, focusing on the appropriateness of treatment and monitoring of drug therapy. Acting as part of the team, individual-level metrics were not collected, only team metrics, which demonstrates the level of the clinic commitment to engaging all providers.

Medication adherence: Adherence to prescribed antiretroviral therapy is essential for maintaining viral suppression in patients with human immunodeficiency virus (HIV) and/or acquired immunodeficiency syndrome (AIDS). Poor adherence is associated with an increased risk of drug resistance, opportunistic infection, virologic failure, hospitalizations, and increased mortality [12,13,14,15,16]. Barriers to medication adherence typically revolve around unmet education and fiscal needs, both of which were identified and addressed through increased, focused provider communication facilitated by embedded pharmacists.

In 2014, a University-sponsored non-profit community pharmacy, was awarded a Ryan White Capacity Grant to create and implement a Patient Centered Pharmacy Program (PCPP) in partnership with a Federally Qualified Health Center (FQHC) to improve HIV management and reduce HIV-related health disparities in rural Idaho. Enrollment, tracking, and delivery forms were created, training materials for staff and patients developed, and unique payment support models (e.g., 340B pricing, enrollment in Idaho’s AIDS Drug Assistance Program, manufacturer coupon cards, and Ryan White Grant) secured by a pharmacy faculty member over a four-month period. Pharmacy students were likewise employed to support training needs, pair individuals with appropriate payers, and foster an environment conducive to support medication adherence that would not have been possible without university and grant subsidization.

Adverse Drug Events: According to the World Health Organization, an adverse drug reaction (ADR) is a “response to a medication that is noxious and unintended” [17]. Many ADRs are preventable, including known side-effects related to medication administration or a drug-drug interaction, but they may be unexpected, such as an allergic reaction. Approximately 3.5% of all hospital admissions are attributed to an ADR, and as the number and complexity of drug therapies increase, the number of ADRs is expected to rise [18,19,20,21,22].

In the current practice environment, primary care providers face increasing demands on their time (e.g., service and authorization requests, documentation demands) and shorter patient visits, resulting in fewer healthcare issues addressed and diminished patient understanding [23,24,25,26]. Time spent on patient education, medication/therapy management, and care coordination is significantly reduced. This reduced time results in a knowledge gap, the “why behind treatment” is unclear and the adverse drug event risk increased, especially in older adults with co-morbid conditions with complex medication regimens [27,28].

In July 2007, an embedded, university-sponsored pharmacist position was established within one of the internal medicine clinics, a clinic responsible for the management of ~1200 mostly older patients per year. The pharmacist works under a Collaborative Practice Agreement (CPA) signed by all the providers (nurse practitioners, physician assistants, and physicians) in the clinic. Patients are referred to the embedded pharmacist for comprehensive medication reviews and chronic disease management of diabetes, hypertension, hyperlipidemia, hyperthyroidism, asthma, and/or chronic obstructive pulmonary disease. A team-based approach to care allows for real-time problem-solving, with healthcare teams working together to make both individual level and program-based care decisions to optimize drug therapy and to prevent ADRs. These co-developed plans decrease inappropriate service utilization, free up providers to focus on their discipline specific scope of practice, and improve health outcomes.

Medication-focused disease management activities that improve adherence and outcomes include medication reconciliation, pre-emptive prior authorization requests, medication substitutions, and ongoing, chronic disease management. Students completing rotations assist with medication reconciliation, chart reviews, and researching drug information questions from providers, staff, and patients, further expanding the impact of the pharmacist within the practice. In 2019, the embedded pharmacists, working two days a week on site, completed 771 in-person and phone visits, notable interventions included but were not limited to removal or addition of medication therapy (n = 85), adjustment of medication dose (n = 235), changing ineffective therapy (n = 24), adherence identification and intervention (n = 58), and patient education (n = 75).

In 2016, an embedded pharmacist position was created at the private medical group senior care clinic (SCC), enabling it to achieve a Patient-Centered Medical Home (PCMH) status. The clinic provider group, comprised of mostly seasoned practitioners, had never worked with an embedded clinical pharmacist and did not know what services could be provided outside of medication reconciliation. Through shadowing, engagement with providers, and collaboration with other PCMH-certified clinics within the SCC providers learned how other facilities across the country were utilizing embedded pharmacists to improve patient care beyond medication reconciliation. Collaborative practice agreements were developed, and the pharmacist scope was expanded to include the referral-based chronic disease state management, with a focus on reduction in documented ADRs and improved medication therapy outcomes.

Despite these advances, sustainability and expansion of the SCC embedded-pharmacy practice was limited by the ability of the pharmacist to bill for the health services provided. The ability of the pharmacist to bill for non-dispensing health services was limited by staff awareness, healthcare facility infrastructure, and supports. A portion of the pharmacist’s time was subsidized by the university to support co-development and pilot testing of a billing and coding toolkit to support training and necessary coding and billing infrastructure within the EHR and facility. Over the past year, processes for submitting claims to both public and private payers have been established, over 100 claims submitted, reasons for rejected claims collected, and patient satisfaction measured.

## 4. Discussion

In this manuscript, we demonstrate how five practice sites approached and implemented non-dispensing pharmacy health services in the primary care setting to enhance medication adherence, improve health outcomes, and reduce the number of adverse events [29,30]. We utilized the CFIR implementation framework to identify, understand, and highlight complex multicomponent healthcare factors that influenced the implementation of embedded pharmacists within the primary care practice.

CFIR allowed researchers across sites to establish a common vernacular to facilitate systematic interventions. We identified two overarching modifiable factors that influence current and future practice delivery and highlight the role of academia as an incubator for practice change and implementation: Data collection and information sharing.

Improved Data Collection: Making a convincing argument that a pharmacist should be added to a care team may first require an objective proof that a pharmacist will add value and help meet the needs of that team. This was the case for each of our sites and became evidence as each site shared their anecdotal and/or limited data during the semi-structured interviews. Data collection grew slowly with the addition of clinical tasks and responsibilities. Initially, pharmacists consulted with providers, reviewing charts, and identifying issues, with little to no documentation of their efforts in the medical record. As the interactions between providers, pharmacists, and patients increased, documentation and collection of data increased. However, current methods of data collection at the embedded sites are onerous (with the exception of the grant funded specialty position), systems are not in place to support collection of pharmacist interventions, and make it difficult to recognize and reimburse individual providers for their contribution. Without a streamlined method to collect and differentiate contributions, it remains difficult for pharmacists to justify the need to expand the clinical services offered.

Pharmacists in many different care settings are tracking their interventions to establish the value they provide and help justify their current roles (as well as new roles). Once established, pharmacists may use intervention tracking to identify opportunities for billing. Though in some settings, these data are preliminary or significantly lacking, leaving a desire for more evidence-based practice data [31,32,33].

Practice-based evidence requires the collection, utilization, and sharing of available data. In order to be sustainable, data processes also need to be efficient. In our case series, we found that data collection strategies varied among practice sites and healthcare systems. To track data, some pharmacists used features of their EHR (e.g., intervention tracking in Epic), self-developed mechanisms (e.g., spreadsheet of interventions), or a mixture of both approaches. Such individual-level approaches make data aggregation, utilization, and sharing difficult. While a universally utilized, nationwide data system for pharmacists (and all healthcare practitioners) is ideal, it is unlikely to occur in the immediate future. However, it is feasible for pharmacists working in the described university system partnership to ensure consistency in data collection, which would allow for greater ease in data aggregation and cross-site comparisons.

With the difficulties in collecting data and the potentially significant time requirements, it is very important to consider what types of data should be collected and shared. Pharmacists should consider what type of activities should be recorded, what level of detail would be required, and what data would best demonstrate the value of the pharmacist/pharmacy team. Organizing interventions into categories can help narrow the focus. Some categories may include clinical care, consultations, cost savings, and patient education. The particular setting, role, and responsibilities of the pharmacist will help determine what data are collected.

To make the goal of tracking pharmacist interventions attainable, the process of recording data needs to be practical [34]. Pharmacists should ask, is there a workflow-based, efficient recording strategy I can utilize? To ensure data is efficiently recorded and adequately captured, an interface with the current software (e.g., current electronic health record) will be important. Furthermore, most pharmacists are not data experts and may require assistance from information technology personnel or those with informatics backgrounds. In future academic/clinical contracts, it may be helpful to ensure that efficient data collection is included. Standardizing data collection methods would also allow pharmacists to collaborate on research more effectively [35].

Improved Data/Information Sharing: Once substantial data are collected, the dissemination of those data to key stakeholders (health system administrators, legislators, other healthcare providers, etc.) both internally and externally is essential for continued advancement of pharmacy integration and practice transformation [1,36,37]. Currently, at all but one of our sites, work-arounds have been created to collect and share information across providers. At the one site grant funds supported data template development, form creation, and data collection. EHR templates that have been adapted from other health professional EHR templates are not linked to other patient information, efforts to collect and share information unnecessarily repeated, and note fields often used inefficiently and ineffectively communicate vital patient health information among team members. In our experience, when data are shared judiciously among team members and across interprofessional teams, it can affect and spur practice change. However, diffusion, dissemination, and implementation of published clinical research results into the practice needs to be more strategic to reach the proposed change.

Internal dissemination of pharmacy interventions and data is varied among practice sites. Stakeholders may value different information depending on the practice site and their role in the organization. Internal data are generally routed to health system administrators such as chief medical officer (CMO), chief operations officer (COO), chief executive officer (CEO), chief financial officer (CFO), quality managers, and other healthcare providers. For example, at our newest practice site, data from pharmacist interventions are routed to the director of pharmacy, who then shares this valuable information with the executive suite which includes the CMO, CFO, and COO. The CMO and COO then communicate relevant findings directly to the medical providers at a monthly staff meeting. The pharmacist attends these monthly staff meetings along with behavioral health, population health, diabetes task force, and the clinical leadership team, which has assisted in integrating the new pharmacist clinical services into the practice site. Similarly, another clinic has quarterly quality meetings in which health metrics such as the percentage of patients with controlled diabetes, hypertension, and hyperlipidemia are reported to the staff. These meetings can be an opportune time to highlight pharmacist interventions and impact on the quality of care.

Despite the significant number of health advances, a substantial gap remains between sharing of this information and resultant incorporation into the clinical practice. Strategic and proactive efforts to improve data sharing are required to facilitate adoption, scale practice change, and optimize patient care delivery. To accomplish this the right information needs to be shared with the right individuals. This can be one of the greatest challenges in external data dissemination. Careful thought must be placed on whether to pursue publication in pharmacy, medicine, or public health journals. Although the amount of pharmacist publications in major medical journals has increased over the past two decades, the amount of published systematic reviews remains lacking. This may be due to the previously mentioned challenge in standardizing data collection methods.

Data from our clinics have primarily been shared in pharmacy journals. Prior to establishing any clinical service, a pharmacist needs to be able to obtain clinical privileges or a scope of practice. Sharing how other pharmacists have done this in the past and possible examples of the scopes included can guide pharmacists attempting to implement new services. However, other key stakeholders such as physicians and health system administrators may not have exposure to these publications and thus may not as widely recognize the impact pharmacists can have on clinical outcomes and quality metrics. In the future, continued expansion of pharmacist publications to major medical journals may assist in the circulation of key findings.

## 5. Conclusions

Academic pharmacists can be used to support program development, expand pharmacist non-dispensing services in primary care, and ultimately serve as incubators for practice change. However, data collection and information sharing are two modifiable factors that need to be addressed to better influence current and future pharmacist practice delivery.

## Figures and Tables

**Table 1:** Select insights on practice readiness: Relationship between CFIR domains, Centers for Medicare and Medicaid Services (CMS) core primary care functions, and embedded pharmacist experiences and perceptions.

CFIR Domain	CPC Components	Embedded Pharmacist Experiences: Practice Site Findings	Embedded Pharmacist Perceptions: Contributing Factors to Readiness
Intervention characteristics	Care Management Processes - Patient outcomes	Practices faced challenges documenting, coding, and billing for services within established EHR systems.	University subsidized pharmacist and student time served as an incubator for trialing processes, expansion of programs, and establishment of evaluation metrics.
	Access and Continuity - Patient outcomes - Medication adherence	Access to educational, training, and grant resources improved incorporation of evidence-based practice in rural communities and improved access to necessary clinical and payment support resources.	University and grant subsidized pharmacist and student time served as an incubator for development and trialing and expanding of clinical pharmacists’ programs for underserved and under-resources communities (e.g., rural residents).
	Planned Chronic and Preventative Care - Adverse drug events - Patient outcomes	Access to educational and training resources improved incorporation of evidence-based practice in rural communities. Better understanding of necessary coding and billing processes were required to provide care and sustain embedded pharmacist service delivery.	University subsidized pharmacist and student time as well as available education and training resources supported embedded pharmacist program management, complex regimen management, and service expansion.
	Patient Engagement - Medication adherence - Patient outcomes	Practice members perceived engagement with patients as vital to improve patient outcomes, self-management, and treatment adherence.	University subsidized pharmacist and student time resulted in increased patient engagement and supported chronic disease self-management.
	Care Coordination - Medication adherence - Patient outcomes - Adverse drug events	Perceived coordination within the patient centered medical home (PCMH) ensured patient follow-up, improved adherence, reduced adverse events, and supported improved patient outcomes.	University subsidized pharmacist and student time reduced clinician burden, improved clinical problem identification, and supported optimization of therapy.
Outer setting	Care Management Processes - Adverse drug events - Medication adherence - Patient outcomes	Effectively and efficiently addressing patient needs was a common challenge, this included personnel time and economic resources.	Time and resources required to meet patient needs were addressed by embedded pharmacists and students in the primary care clinic. Connecting individuals with medication assistance programs and grant supports.
	Access and Continuity	Site health information technology systems and billing infrastructure were unable to support state documentation, coding, and billing requirements required for service provision and sustainability.	Sharing of technology resources, EHR templates, CPAs, policies, and training materials across sites.
	Patient Engagement - Patient outcomes - Medication adherence	Help patients in rural and underserved communities manage chronic health conditions and access necessary supports.	Using the PCMH model, patients in rural and underserved communities received more attention from the healthcare system.
Inner setting	Care Management Processes - Adverse drug events - Patient outcomes	Health information technology systems and billing infrastructure were unable to support documentation, coding, and billing requirements to support service provision, expansion of services, and/or sustainability.	University subsidized pharmacist and grant funding to support training, infrastructure development, advocacy, and improve EHR and billing infrastructure.
	Access and Continuity - Patient outcomes	Embedded pharmacists and students often functioned on a consultant basis and were not present within the primary care clinic due to teaching requirements, resulting in limited access, missed interventions, and sporadic engagement with the team.	Practices with embedded pharmacists’ offices centrally located were more accessible to providers and patients and utilized more by the PCMH team.
Characteristics of individuals	Care Management Processes - Adverse drug events - Patient outcomes - Medication adherence	Primary care provider groups who worked with embedded pharmacists in the past were more likely to work with pharmacists and students to improve/optimize drug therapy.	Primary care practices that worked with embedded pharmacists to establish and modify workflows were more likely to successfully impact patient outcomes, improve medication adherence, identify and reduce adverse drug events.
	Access and Continuity - Adverse drug events - Patient outcomes	Practices that believed in the value of embedded pharmacists worked with healthcare systems to incorporate them into the PCMH and daily practice.	Practices tended to utilize embedded pharmacists and students that were physically available, that rounded with the team, and that participated in clinical activities (e.g., lunch and learns, grand rounds).
	Patient Engagement - Patient outcomes	Patients and staff tend to reach out to individuals with similar values and beliefs.	Healthcare administrators and university providers supported students and pharmacists with whom they have relationships, shared mission, vision, and clinical practice goals.
Implementation process	Care Management Processes - Adverse drug events - Patient outcomes	Shared goals, consistent vernacular and tailored training and supports were required for sustainable practice site improvements.	Co-development of policies, procedures, and workflows is required. Consistent and comprehensive informal and formal training with opportunities for hands-on practice is required for program implementation and sustainability.
	Patient Engagement	Engagement of patients, staff, and providers were required to sustain practice-level improvements.	Healthcare administrators and university providers supported students and pharmacists that add value to the organizations. This is further strengthened when the service is valued by external partners (e.g., insurers).

**Table 2:** Identified facilitators and barriers to implementation across sites.

IMPLEMENTATION (Embedded Pharmacist Position, Family Practice Clinic)				
Characteristics of Intervention ^a^	Inner Setting	Outer Setting	Individuals Involved	Implementation Process
- Subsidized position (university sponsored) - Academic incubator - Connection with educational and training resources - Program expansion	- Physician-owned clinic - Office on site - For profit - Teaching facility for Rx students - Barriers to provider understanding	- Community support - Incentives for students - Unmet patient needs - Provider time constraints	- Pharmacist(s) at site - Intern/Students - Patients (self-efficacy) - Providers (diverse) - University system (Rx)	- Workflow development (enrollment, tracking, delivery) - Engaging with community - Engaging with providers - Educating providers and staff
**IMPLEMENTATION (Embedded Pharmacist Position, Internal Medicine Clinic)**				
**Characteristics of Intervention**	**Inner Setting**	**Outer Setting**	**Individuals Involved**	**Implementation Process**
- Subsidized (university sponsored) - Academic incubator - Connection with educational and training resources - Program expansion - Management services offered: Diabetes, hypertension, comprehensive medication reviews	- Primary care clinic - Office on site - Teaching facility for Rx students	- Community support - Incentives for students - Unmet patient needs - Provider time constraints - Larger organization, additional authorization (change slow)	- Pharmacist at site - Intern/Students - Patients (self-efficacy) - Providers (diverse) - University system (Rx) - Healthcare administration - Information technology (IT) - Billing/Finance	- Workflow development (enrollment, tracking, delivery) - Engaging with community - Engaging with providers - Educating providers and staff - Readiness for implementation (IT billing, and finance lagged behind)
**IMPLEMENTATION (Embedded Pharmacist Position, Rural Health)**				
**Characteristics of Intervention**	**Inner Setting**	**Outer Setting**	**Individuals Involved**	**Implementation Process**
- Subsidized (university sponsored) - Academic incubator - Connection with educational and training resources - Growing complexity and program expansion	- Primary care clinic (MD owned until 2016) - Office on site - Teaching facility for Rx students	- Community support - Incentives for students - Unmet patient needs - Provider time constraints	- Pharmacist at site - Intern/Students - Patients (self-efficacy) - Providers (diverse) - University system	- Workflow (consultant role) - Engaging with community - Engaging with providers - Educating providers and staff (formal and informal)
**IMPLEMENTATION (HIV/AIDs Incubator Project at Community Pharmacy)**				
**Characteristics of Intervention**	**Inner Setting**	**Outer Setting**	**Individuals Involved**	**Implementation Process**
- Subsidized (university sponsored) - Academic incubator - Connection with educational and training resources - Grant funded project (metrics mandated)	- Federally qualified health center (FQHC) - Community Pharmacy - Office on site - Teaching facility for Rx students	- Community support - Unmet patient needs - Provider time constraints - External incentives to participate	- Pharmacist(s) at site - Intern/Students - Patients (incentivized, high resource needs) - University system	- Workflow development (enrollment, tracking, delivery) - Engaging with HIV/AIDs community - Training of staff and patients
**IMPLEMENTATION (Embedded Pharmacist Position with Private Medical Group)**				
**Characteristics of Intervention**	**Inner Setting**	**Outer Setting**	**Individuals Involved**	**Implementation Process**
- Subsidized (university sponsored) - Academic incubator for billing - Connection with educational and training resources - Management services offered: Chronic disease management - Healthcare initiated and supported	- Primary care clinic - Office on site - Teaching facility for Rx students	- Community support - Incentives for students - Unmet patient needs - Provider time constraints - External billing	- Pharmacist(s) at site - Intern/Students - Providers (diverse) - University system - Healthcare administration - Information technology (IT) - Billing/Finance	- Workflow development (enrollment, tracking, delivery) - Educating providers and staff - Readiness for implementation (IT, billing and finance lagged behind) - Educating legal (fear of fraud)

^a^ Intervention refers to the addition of the embedded pharmacists within the primary care clinic setting.
